# Lysines Acetylome and Methylome Profiling of H3 and H4 Histones in Trichostatin A—Treated Stem Cells

**DOI:** 10.3390/ijms22042063

**Published:** 2021-02-19

**Authors:** Flora Cozzolino, Ilaria Iacobucci, Vittoria Monaco, Tiziana Angrisano, Maria Monti

**Affiliations:** 1 Department of Chemical Sciences, University Federico II of Naples, Strada Comunale Cinthia, 26, 80126 Naples, Italy; flora.cozzolino@unina.it (F.C.); ilaria.iacobucci@unina.it (I.I.); 2 CEINGE Advanced Biotechnologies, Via G. Salvatore 486, 80145 Naples, Italy; monacovi@ceinge.unina.it; 3 Interuniversity Consortium National Institute of Biostructures and Biosystems (INBB), Viale Medaglie d’Oro, 305-00136 Roma, Italy; 4 Department of Biology, University Federico II of Naples, Strada Comunale Cinthia, 21, 80126 Naples, Italy; tangrisa@unina.it

**Keywords:** histone PTMs, limited proteolysis, mass spectrometry, TSA, activation of differentiation

## Abstract

Trichostatin A ([R-(E,E)]-7-[4-(dimethylamino) phenyl]-N-hydroxy- 4,6-dimethyl- 7-oxo-2,4-heptadienamide, TSA) affects chromatin state through its potent histone deacetylase inhibitory activity. Interfering with the removal of acetyl groups from lysine residues in histones is one of many epigenetic regulatory processes that control gene expression. Histone deacetylase inhibition drives cells toward the differentiation stage, favoring the activation of specific genes. In this paper, we investigated the effects of TSA on H3 and H4 lysine acetylome and methylome profiling in mice embryonic stem cells (ES14), treated with trichostatin A (TSA) by using a new, untargeted approach, consisting of trypsin-limited proteolysis experiments coupled with MALDI-MS and LC-MS/MS analyses. The method was firstly set up on standard chicken core histones to probe the optimized conditions in terms of enzyme:substrate (E:S) ratio and time of proteolysis and, then, applied to investigate the global variations of the acetylation and methylation state of lysine residues of H3 and H4 histone in the embryonic stem cells (ES14) stimulated by TSA and addressed to differentiation. The proposed strategy was found in its simplicity to be extremely effective in achieving the identification and relative quantification of some of the most significant epigenetic modifications, such as acetylation and lysine methylation. Therefore, we believe that it can be used with equal success in wider studies concerning the characterization of all epigenetic modifications.

## 1. Introduction

Trichostatin A ([R-(E,E)]-7-[4-(dimethylamino) phenyl]-N-hydroxy- 4,6-dimethyl- 7-oxo-2,4-heptadienamide, TSA) is a pharmacological agent endowed with potent histone deacetylase inhibitory activity (HDI or HDACI), thus promoting histone acetylation with broad effects on epigenetic signature [1].

TSA-mediated HDAC inhibition contributes to chromatin relaxation, allowing transcription factors to access the DNA molecule within the chromatin structure. TSA was also reported to promote morphology and gene expression changes in embryonic stem cells (ESCs) [2]. ESCs cultured in DMSO form round and compact colonies, while after TSA treatment, colonies are disrupted, and cells become flattened [3]. Inhibition of histone deacetylases drives cells toward differentiation by regulating the expression of differentiation- and pluripotency-associated genes. Many studies demonstrated that TSA enhances cell differentiation [4,5,6] by negatively regulating the expression of stemness markers [7], such as *Oct4* [8], and simultaneously activating differentiation markers, such as *Pdx1* [9]. The molecular mechanism of TSA-mediated regulation of gene activation and repression is very likely related to post-translational modifications of histones. Interfering with the removal of acetyl groups from lysine residues in histones is one of many epigenetic regulatory processes that control gene expression. Because of its biochemical activities, TSA has some activities as an anticancer drug [10,11] or as a cell differentiation promoter [12].

It is well known that lysine acetylation or methylation of various residues on histone H3 and H4 are involved in transcriptional activation and/or silencing of different genes. As a consequence, the combination of all modifications occurring within a histone, in a nucleosome, and, in turn, within different nucleosomes, finely promotes the recruitment of various components of multiprotein complexes, including a large number of enzymes and accessories proteins known as writers, readers, and erasers [13], whose presence and activities depend on specific molecular stimuli [14].

The qualitative and quantitative investigation of histone post-translational modification (PTM) has been approached with several methods, most of them based on the employment of specific antibodies [15]. However, all antibody-based techniques are target specific, and it is necessary to know the position and type of modification of interest ab initio. More recently, untargeted alternative strategies have been developed for the identification, localization, and quantification of epigenetic PTMs [16], among which the most promising rely on the employment of mass spectrometry (MS) methodologies, essentially following mass-mapping-based approaches [17,18]. Despite the advantages of mass spectrometry technology in terms of versatility and sensitivity, two main problems still remain to be solved entirely. They concern the development of suitable proteolytic digestion protocols and the high level of PTM heterogeneity in the sample to be analyzed. Mass-mapping approaches rely on the use of proteolytic enzymes, above all trypsin, commonly used in all proteomic applications. Unfortunately, the very high number of basic residues occurring within the small histone proteins leads to the formation of short peptides, impairing an effective MS analysis. Although alternative enzymatic digestion protocols have been tested [19,20,21,22], this drawback still remains challenging. Moreover, PTM heterogeneity generates isobaric peptides containing the same modification located at different positions along the peptide chain, making their localization very difficult [23].

This paper reports a new approach for investigating histone epigenetic modifications based on stringent trypsin digestion conditions to control hydrolysis kinetics and generate peptides of suitable length for MS analysis. The identification and localization of occurring PTMs relied on peptide retention times and fragmentation spectra recorded in LC-MS/MS analyses. The whole procedure was developed on the standard chicken core histones by testing short incubation time and different enzyme to substrate (E:S) ratios to achieve the highest sequence coverage for H3 and H4 histones. The optimized protocol was then applied for a proteome-wide investigation of the acetylation and methylation states of histone H3 and H4 in ES14 mouse cells following either TSA or DMSO treatment. The established strategy led to the identification and relative quantification of H3 and H4 acetylation/methylation distribution in differentiating (TSA treated) and stemness-retained (DMSO incubation) ES14 mouse cells.

## 2. Results

### 2.1. TSA Induces a Change of Morphology Corresponding to Oct4 and Pdx1 Gene Regulation in ESCs

**Figure 1:**
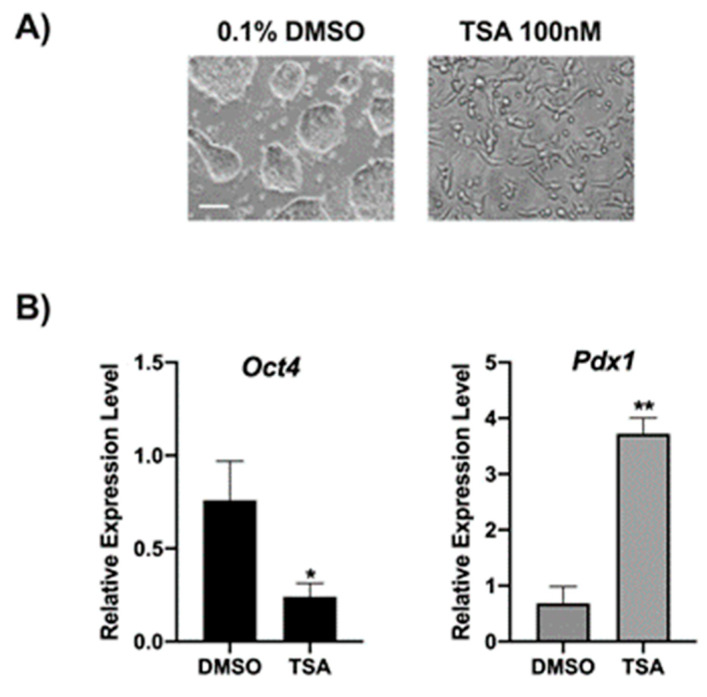
Trichostatin A (TSA) induces differentiation in ES14. (**A**) Phase-contrast microscopy analysis of ES14 cells following treatment with 0.1% DMSO or 100 nM TSA clearly showing morphology changes (scale bar, 100 µm). (**B**) Gene expression analysis by qPCR for *Oct4* (left panel) and *Pdx1* (right panel) genes showing that incubation with TSA drives ES14 cells toward differentiation. Data points represent the average of triplicate determinations ± SD. Similar results were obtained in three independent experiments. * *p* < 0.05; ** *p* < 0.01.

In order to validate our experimental system, the morphology of ES14 cells treated with either TSA or DMSO for 24 h was analyzed by phase-contrast microscopy. Figure 1A shows that ESCs treated with TSA undergo morphological changes suggestive of differentiation. Moreover, the expression of *Oct4* and *Pdx1* genes, markers of stemness and differentiation, respectively, was evaluated by qPCR. Data revealed a significant decrease of *Oct4* expression (Figure 1B, left panel) instead of the increased expression of *Pdx1* (Figure 1B, right panel), confirming the tendency of TSA to induce differentiation and validating the experimental system.

### 2.2. Limited Proteolysis Standardization of H4 and H3 Histones

**Figure 2:**
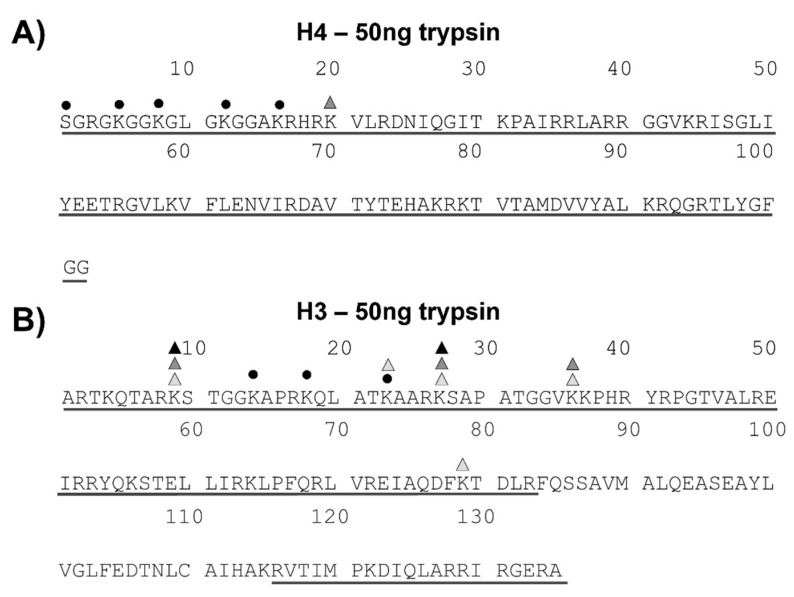
MALDI-MS- and LC-MS/MS-derived histone mass mapping with the identified modifications. The underlined regions represent the covered sequences obtained by MALDI-MS and/or by LC-MS/MS analyses. Black circles represent acetylation. Light grey, dark grey, and black triangles are for methylation, di-methylation, and tri-methylation, respectively. (**A**) Histone H4 mass mapping obtained by incubating 4 µg of chicken core histones with 50 ng of trypsin in the limited proteolysis protocol. (**B**) Histone H3 mapping obtained from the reaction of 4 µg of chicken core histones with 50 ng of trypsin in the limited proteolysis procedure.

H3 and H4 histones were isolated from 4 µg of a standard mixture of chicken core histones following SDS-PAGE fractionation. Protein bands corresponding to H3 and H4 species were excised from the gel and in situ digested with either 100, 50, or 10 ng of trypsin in 50 mM ammonium bicarbonate for 2 h. The tryptic peptides released from each enzymatic digestion were analyzed by MALDI-MS and LC-MS/MS and mapped onto the respective H3 and H4 sequences. Mass spectral data showed that the tryptic peptides obtained using 50 ng of trypsin were suitable in size for effective MS and MS/MS analyses and led to the highest sequence coverage for both H4 (100%) and H3 (83%) proteins (Figure 2).

Supplementary Table S1 reports the H4 (Figure 2A) and H3 (Figure 2B) tryptic peptides identified by MS analyses in the best digestion conditions. Furthermore, MS experiments revealed the occurrence of several post-translation modifications, including multiple acetylations (ΔM = +n42 Da) and methylations (ΔM = +n14 Da).

In H4, the N-terminal peptides 1–12, 1–16, 1–17, and 18–36 were found largely and heterogeneously modified (Supplementary Table S1A). A careful inspection of the MS/MS spectra of modified peptides identified the presence of acetylation sites at the level of the N-terminus, K5, K8, K12, and K16, and the occurrence of di-methylation at K20 (Figure 2A), according to literature data [24]. This analysis also confirmed that multiply acetylated peptides consists of a heterogeneous mixture of isobaric species carrying the acetyl groups on different lysine residues. Supplementary Table S1B reports the peptides identified in the H3 tryptic digest obtained by incubation with 50 ng of trypsin. Although the H3 sequence coverage was not complete, the N-terminal 83 amino acids portion, i.e., the H3 region where most PTMs occur, was completely mapped. Again, several post-translational modifications were detected in the H3 sequence and allocated by LC-MS/MS analysis. Acetylation was detected at K14, K18, and K23. In addition, several methylated residues were also found: mono-methylation was pinpointed at K9, K27, K36, and K79, and both di- and tri-methylation were identified at K9 and K27 by manual inspection of the MS and MS/MS spectra confirming literature data (Figure 2B) [25].

### 2.3. Analysis of H4 Acetylation Landscape in DMSO vs. TSA

The different degrees of acetylation in histones H4 and H3 from embryonic stem cells ES14 during differentiation were evaluated using the methodology described above. In addition, extracted ion chromatogram (XIC) procedures based on the evaluation of the area of chromatographic peaks associated with the peptide ions extracted from the total chromatogram were employed for relative quantification of the differently modified peptides. The chromatographic areas of the H4 46–55 and H3 57–63 unmodified peptide ions were used for the normalization of H4 and H3 data, respectively (Supplementary Table S2A,B). Both these peptides were chosen as internal standards since they are located in unmodified protein regions and are then present in homogeneous form.

ES14 cells were allowed to differentiate by treatment with TSA, while the undifferentiated state was maintained by incubation with DMSO and used as control. Cells grown in DMSO or treated with TSA were lysed, and the histone proteins were extracted in acidic conditions and fractionated by SDS-PAGE. Protein bands corresponding to H4 and H3 histones were quantified by densitometric analysis and treated with a suitable amount of trypsin under strictly controlled conditions. The resulting tryptic peptide mixtures were analyzed by both MALDI-MS and LC-MS/MS.

**Figure 3:**
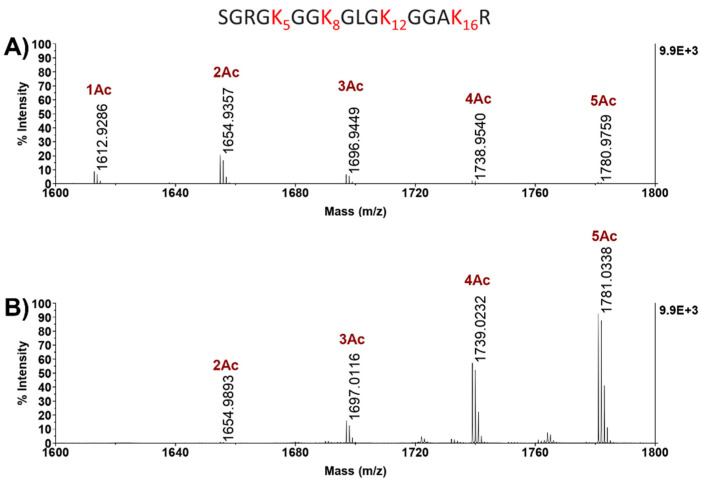
Partial histone H4 MALDI-MS spectra of E14 ES mouse treated cells. *m*/*z* signals of differently modified species relative to peptide 1–17 are highlighted in DMSO- (**A**), and TSA-treated cells (**B**). Peptide 1–17 sequence is reported upper in figure; in red K residues are highlighted, as potential targets of acetylation, in addition to the N-terminus. Ac: acetyl group. The spectra are reported in the same scale of intensity.

Figure 3 shows the partial MALDI-MS spectra of tryptic peptides from histone H4 extracted from DMSO- (Figure 3A) and TSA (Figure 3B)-treated ES14 cells in the *m*/*z* region where the signals corresponding to the differently modified forms of the 1–17 peptide were recorded. As expected, both in DMSO and in TSA cells, H4 is multi-acetylated in the N-terminal portion where, besides the N-terminus, four other acetylation sites are present at K5, K8, K12, and K16. However, some differences in the two samples could easily be observed.

Although the MALDI-MS analysis does not allow a quantitative evaluation of peptides, a general increase in acetylated species was observed in the presence of TSA with an evident change in the relative abundance of the differently acetylated components. In the DMSO sample, the di-acetylated form of the 1–17 peptide was the main species, while species bearing four and five acetyl groups were barely detectable. On the contrary, in the TSA sample, the fully acetylated peptides 1–17 carrying five acetyl groups was the most abundant species, with the mono- and di-acetylated species being almost undetectable.

**Figure 4:**
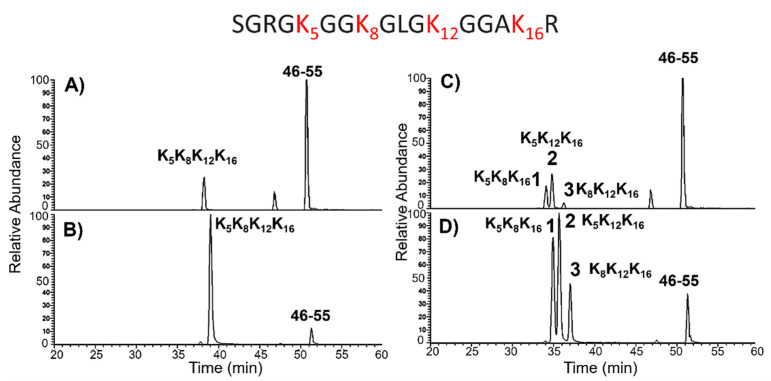
Extracted ion chromatogram (XIC) of selected peptides derived from histone H4 E14 ES mouse treated cells. The doubly charged 890.99 *m*/*z* signal corresponding to peptides 1–17 bearing five acetyl groups and reference peptides 46–55 are reported in DMSO- (**A**), and TSA (**B**)-treated cells. The 869.99 *m*/*z* values relative to doubly charged peptides 1–17 carrying four acetyl groups are reported. (**C**) Extracted ion chromatogram recorded for DMSO-treated sample. (**D**) Extracted ion chromatograms recorded for TSA-treated in TSA K5K8K16 (RT: 34.94 min), K5K12K16 (RT: 35.67 min), K8K12K16 (RT: 37.05 min) are detected as separate forms in TSA-grown cells. N-terminus is always modified by acetyl group, and reference peptide is also reported. Peptide 1–17 sequence is reported upper in figure; in red K residues are highlighted, as potential targets of acetylation, in addition to the N-terminus. Ac: acetyl group.

Figure 4A,B shows the XIC chromatograms of the fully acetylated peptides 1–17 bearing five acetyl groups (N-terminus, K5, K8, K12, and K16) and the reference peptides 46–55 from DMSO- (Figure 4A) and TSA (Figure 4B)-treated ES14 cells. Relative quantification of the fully acetylated (1–17) peptide in the two conditions was performed by comparing the XIC area of the corresponding peptide ions in both states after normalization with the area of the 46–55 reference peptide ion. The calculated ratio was 66.3 (Table 1), confirming the occurrence of a higher amount of the fully acetylated form in the TSA-treated cells, as suggested by MALDI-MS analysis (Figure 3).

As stated above, multi-acetylated peptides showing the same mass might consist of a heterogeneous mixture of isobaric species displaying a different distribution of modifying groups that are indistinguishable in MALDI-MS analysis. The H4 tryptic digest was then analyzed by LC-MS/MS, and both the modification sites and the relative abundance of the modified species were assigned based on their retention time and fragmentation spectra following the XIC procedure. When the partially acetylated forms of the 1–17 peptide were analyzed, a complete resolution and identification of the isobaric species could be obtained. As an example, Figure 4C,D shows the XIC chromatograms of the ion at *m*/*z* 869.99 corresponding to the doubly charged peptides 1–17 carrying four acetyl groups in DMSO- (Figure 4C), and TSA (Figure 4D)-treated ES14 cells. In both conditions, the modified peptide originated three isobaric species differing in the distribution of the acetylated residues that could be distinguished by their different retention times (Peaks 1, 2, and 3 in Figure 4C,D).

**Figure 5:**
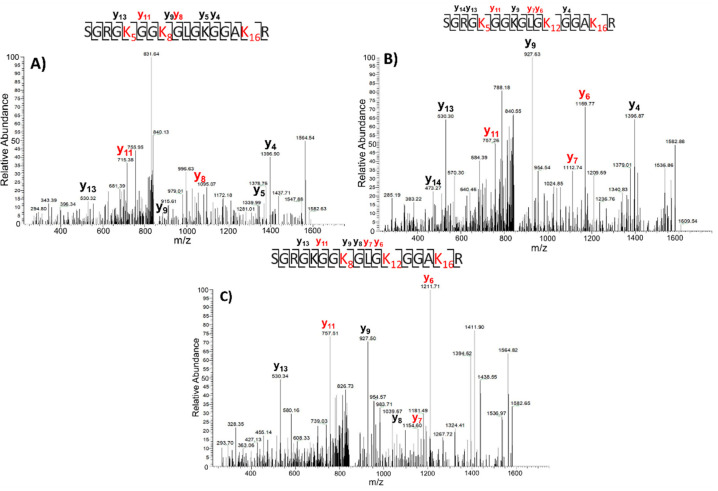
Fragmentation spectra of 1–17 peptide carrying four acetyl groups (including the N-terminus) in TSA-grown cells of (**A**) Peak 1 relative to K5acK8acK16ac (RT: 34.94 min), (**B**) peak 2 relative to K5acK12acK16ac (RT: 35.67 min), and (**C**) peak 3 relative to K8acK12acK16ac (RT: 37.05 min). In red, the differing daughter ions are highlighted.

According to their fragmentation spectra, the three species could easily be identified. Peak 1 corresponds to the peptide acetylated at the N-terminus, K5, K8, and K16. Peak 2 was associated with the peptide carrying acetyl groups at the N-terminus, K5, K12, and K16; and peak 3 consists in the 1–17 peptide bearing acetylation at the N-terminus K8, K12, and K16. The fragmentation spectra of Peak 1, 2, and 3 from the TSA sample are reported in Figure 5.

The XIC chromatograms were then used to evaluate the different abundance of each species in the two conditions using the area of the 46–55 peptide ion (Figure 4C,D) as an internal reference for normalization. Again, a general increase in the amount of acetylated species in the presence of TSA was observed. However, some differences could be detected in the three isobaric species. When the normalized areas of the three peaks were compared in the two conditions, Peak 1 increased by about 24 times in the TSA sample, Peak 2 by about 21 times, and Peak 3 by about 54 times. Therefore, besides a general increase in the acetylated level, the TSA treatment induces a higher effect on the fragment bearing acetyl groups at K8, K12, and K16.

Moreover, according to preliminary MALDI-MS analyses, the low acetylated components, i.e., the mono- and di-acetylated peptides, are the least abundant species in the TSA sample.

### 2.4. Analysis of H3 Acetylation Landscape in DMSO vs. TSA

The same procedure was also applied to investigate the acetylation and methylation state of histone H3 during TSA-induced differentiation. Differing from H4, histone H3 is reported to be largely subjected to methyl modifications [26], and then, besides the effect of acetylation, we also evaluated TSA’s indirect effect on H3 methylation patterns. In this particular case, MALDI-MS analyses were not sufficient since the increase in molecular weight associated with tri-methylation of lysine residues (+42 Da) is not distinguishable from acetylation in MALDI-MS spectra. H3 samples from TSA- and DMSO-treated ES14 cells were digested under strictly controlled conditions, and the resulting peptide mixtures were analyzed by LC-MS/MS on a high-resolution mass spectrometer to uniquely identify, localize, and quantify each specific modified species.

**Figure 6:**
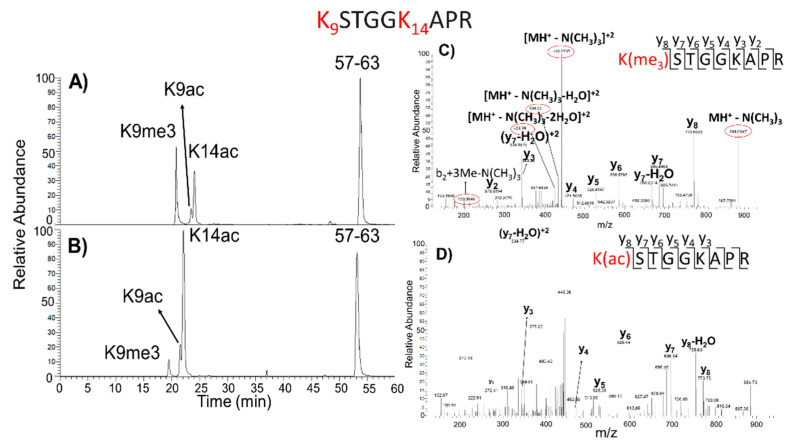
Extracted ion chromatogram (XIC) of tryptic peptide ion 9–17 of H3 histone from E14 mouse ES cells treated with DMSO (**A**) or TSA (**B**). Peak derived from the doubly charged 472.2867 *m*/*z* was detected as the species carrying a trimethylation on K9 (lowest RT), 472.2705 *m*/*z* corresponded to the acetyl group on K9 (middle RT), 472.2687 *m*/*z* was relative to K14 acetylated (highest RT), and reference peptides 57–63 eluting with an RT about 53 min is also reported. The fragmentation spectra of tri-methylated (Me3) (**C**) and acetylated species (**D**) are also reported. Peptide 9–17 sequence is reported upper in figure; in red K residues are highlighted, as potential targets of PTMs. In red circles, the specific daughter ions are highlighted. Me: methyl group; Ac: acetyl group.

Data recorded in the LC-MS/MS analyses were effective for distinguishing the isobaric forms of the modified peptides. For example, the mono-acetylated and singly tri-methylated species of the H3 fragment 9–17 are discussed in detail. Peptides 9–17, containing two lysine residues (K9 and K14), generated three isobaric species showing ΔM = +42 Da and a nominal mass of 943 Da, which were easily separated by reverse-phase chromatography. Figure 6A,B shows the corresponding XIC chromatograms, in which the three peaks were distinguishable in both conditions, according to their retention times and specific fragmentation spectra. The peak with the lowest retention time (20.74 min) in DMSO and TSA samples was assigned to the tri-methylated peptides 9–17 by its specific fragmentation spectra, reported in Figure 6C, in which diagnostic peaks assigned to tri-methyl lysine 9 are circled in red. On the other hand, the fragmentation spectrum of second peak at 23.47 min (Figure 6D) showed the occurrence of daughter ions relative to acetyl K9. Finally, the peak at the highest retention times (24.06 min in the DMSO and 22.07 min in TSA samples) was assigned to the peptide ion 9–17 acetylated at K14 (data not shown). This lysine was never found as a tri-methylated residue.

Once the identity of the isobaric peaks was established, their relative abundance in the two conditions was calculated according to the XIC procedure, using the 57–63 peptide ion area for normalization. The species carrying tri-methylated K9 was found to be about six times decreased in TSA-treated samples. In contrast, the mono-acetylated components on K9 and K14 were about two- and threefold higher, respectively, suggesting that TSA treatment increased acetylation at K9, with a corresponding decrease of tri-methylation at the same residue.

Measurement of the fully acetylated 9–17 peptide showed a 28-fold increase in the TSA sample in respect to DMSO-treated cells. A summary of the results obtained on 9–17 peptide is given in Table 2.

The same outcome was observed for the 18–26 peptide doubly acetylated at K18 and K23, which exhibited a 13-fold increase under differentiation conditions, i.e., in the presence of TSA-, as compared to the DMSO-treated cells. This effect of TSA promoting multiple acetylation species is in agreement with that observed for H4.

## 3. Discussion

Besides its role in genome packaging, chromatin has a highly dynamic nature in which histones’ post-translational modifications play an active role in modulating gene activation and expression. In this study, we examined lysine residues’ acetylation and methylation global profiles of H3 and H4 histones from E14 mouse embryonic stem cells treated with either trichostatin A (TSA) or dimethylsulfoxide (DMSO) by using a newly developed limited-proteolysis mass-spectrometry protocol. E14 mouse embryonic stem cells are well-characterized and widely used, representing a good model for genome-wide studies, recently also by next-generation sequencing analysis [27].

Trichostatin A is reported to be a cell differentiation promoter, mainly acting as a histone deacetylase inhibitor. The acetylation of histone, mostly H3 and H4, partially neutralizes the positive charges of lysine residues, decreasing their electrostatic interaction with DNA phosphate groups, relaxing chromatin, and allowing the recruitment of the transcriptional machinery for the activation of gene expression [28,29]. The global effects induced by TSA treatment on lysine acetylation and methylation in H3 and H4 has not been investigated in detail. More generally, studying histone PTMs is essentially a challenging task due to the high content of basic amino acids that, following tryptic digestion, originate many short peptides not amenable to mass spectrometry analyses. We developed an optimized procedure based on limited-proteolysis mass spectrometry to identify and quantify H4 and H3 PTMs using standard chicken core histone as a model. This procedure effectively produced tryptic peptides suitable in size for MS analyses and provided high sequence coverage of the two histone proteins.

Once optimized, the protocol was applied to investigate lysine acetylation and methylation profiles in H3 and H4 histones extracted from ES14 cells treated with either TSA or DMSO as control. In agreement with the biological role of TSA, a general increase in the highly acetylated forms of H3 and H4 histones following TSA treatment was observed with an accumulation of the multiple-acetylated peptides. The fully acetylated H4 N-terminal peptides 1–17 bearing five acetyl groups and the doubly acetylated 9–17 species from H3 showed a large increase in the acetylation state, up to 66-fold in H4 and 28-fold in H3. This trend was confirmed by the decrease in the acetylation level of the less-modified species. In H4, the relative abundance of the mono-acetylated peptide at K8 or K16 decreased in TSA cells in respect to DMSO, while acetylation at K12 was roughly unchanged. Similarly, in H3, the amount of the 18–26 peptide carrying a single acetylation at K18 or K23 was lower in TSA than in DMSO, while the di-acetylated species with both residues modified increased by about 13 times in TSA-treated cells.

A detailed investigation of multiple modified peptides confirmed that they consist of a heterogeneous mixture of isobaric species carrying the modifying groups at different lysine residues. Moreover, tri-methylated or mono-acetylated lysine residues exhibited an identical mass difference (+42 Da), making their identification challenging. In these cases, MALDI-MS analyses could not be used; however, LC-MS/MS procedures were instrumental to uniquely identify, localize, and quantify each specific modified species. For example, the three modified forms of the H4 1–17 peptide bearing three acetyl groups and the three isobaric species of the doubly acetylated H3 9–17 peptide could be easily separated, identified, and their relative abundance measured. Analogously, the tri-methylated peptides 9–17 from H3 was distinguished from the mono-acetylated form by its specific fragmentation spectra.

Measurement of the relative abundance of H4 isobaric species revealed that TSA treatment induced different effects on the modified peptides bearing acetyl groups at K5, K8, K12, and K16 that exhibited different fold change upon incubation with the cell differentiation promoter. Moreover, quantification of the tri-methylated H3 peptide demonstrated that methylation of histone residues was also affected by TSA treatment, as increasing acetylation at K9 provided a corresponding decrease of tri-methylation at the same residue.

These findings suggest that TSA inhibition on HDAC activities elicits a concerted effect on the global acetylation within each histone molecule, more than affecting the acetylation state of a single lysine residue. It is well known that the combination of histone modification is a critical factor in gene expression regulation [30]. Several targeted mutagenesis experiments on lysine residues 5, 8, and 12 in H4 showed that changes in gene expression levels are due to cumulative rather than single effects [31]. Moreover, acetylation and deacetylation processes carried out by HATs (histone acetyltransferases) and HDACs (histone deacetylases), respectively, are concerted and finely tuned during the cell cycle and differentiation [32]. According to our findings, the synergy of K8, K12, K16 has been previously described preponderantly in SUM159 and MCF7 cells treated with butyrate, another HDAC inhibitor. Furthermore, a progressive and hierarchical order of H4 acetylation following K20 di-methylation was observed; moreover, K5 was found to be the last lysine acetylated by HATs and the first deacetylated by HDACs [33].

Data retrieved on H3 show the exclusive acetylation of K4 and K27 in the TSA sample. Guillemette et al. found that H3K4Ac modification enriched promoters of actively transcribed genes in *Saccharomyces cerevisiae* and human cells by ChIP experiments [34], while Sato and colleagues observed an H3K27Ac accumulation in the first stage of zebrafish embryo development [35]. These observations might indicate the co-occurrence of specific molecular signaling at the level of individual lysine residues for chromatin relaxation.

Finally, our data on H3 suggested an indirect effect of TSA on lysine methylation, a further hallmark of gene expression regulation. H3K4Me3 and H3K9Me3 are indicated as the signature of transcription activation and repression, respectively, in human cells. Mono-, di-, and tri-methylated K9 were reported as gene silencing marks [36,37,38] and were found downregulated in TSA treated cells. Crosstalk between different modifications of histone Lys residues is well documented. Recently, Wu et al. demonstrated that dimethylation at H3K4me2 occurs more slowly when the adjacent K14 is acetylated, and this residue was found particularly resistant to deacetylation in agreement with our results [39]. Moreover, it was demonstrated that in vivo, several multiprotein complexes such as CoREST NuRD, MiDAC, and the Sin3a complex are composed of different enzymatic players affecting epigenetic modifications [40,41].

Altogether identification and relative quantification of H4 and H3 modifications suggest that TSA treatment induced hyperacetylation of Lys residues, with a concomitant effect also on methylation. These findings might shed further light on the cryptic message of the “histone code” as a global effect of crosstalk between all modifications and the other epigenetic players.

Finally, the results presented in this work showed the efficacy of the newly developed limited proteolysis-mass spectrometry protocol to generate suitable tryptic peptides providing an effective and general strategy to investigate a large number of different histone PTMs.

## 4. Conclusions

In conclusion, this work has presented a new MS-based strategy to investigate histone PTMs. The protocol has been applied in the study of histone acetylation and methylation profiles of E14 mouse embryonic stem cells either grown in TSA- or DMSO-enriched media. The results showed the efficacy of the protocol in the generation of suitable tryptic peptides with an optimized, in-gel, limited proteolysis approach. This procedure effectively produced tryptic peptides suitable in size for MS analyses and can be successfully applied in the investigation of different epigenetic PTMs. In TSA-treated cells, the identification and relative quantification of H4 and H3 histone acetylated and methylated peptides have revealed the largest increase of hyperacetylated species, consisting of globally active chromatin hallmarks.

## 5. Materials and Methods

### 5.1. Standardization of In Situ Limited Proteolysis Protocol

The set up of limited proteolysis conditions on histone H3 and H4 was carried out on standard Core Histones from chicken (Merck Millipore, US-MA). Briefly, 4 µg of core histones were loaded on a 15% acrylamide/bis-acrylamide SDS-PAGE gel (Supplementary Figure S1). The bands corresponding to H3 and H4 were cut, and in situ digestions were performed by using different trypsin (Sigma Aldrich, St. Louis, Missouri, USA) amounts (10, 50, or 100 ng of trypsin/band) and a short incubation time (2 h) to obtain peptides of appropriate length for MS analyses and the highest histone sequence coverage. In-gel hydrolyses were carried out in ammonium bicarbonate 50 mM. Peptides were extracted in acetonitrile and formic acid 0.2% and vacuum dried with a SpeedVac system.

### 5.2. Mass Spectrometry Analyses and Relative Quantification

Mass spectrometry techniques were employed to analyze histone peptide mixtures obtained from in situ hydrolysis. Particularly, MALDI-TOF analyses were performed using a MALDI-TOF/TOF 4800 (AB-SCIEX) mixing 1:1 samples with 10 mg/mL α-cyano-4-hydroxycinnamic acid matrix. Samples were dissolved in a 2% TFA matrix in 70% acetonitrile, 30% TFA 0.2%. Instrument setup was positive reflectron mode; MS acquiring range was set up from 600 to 5000 *m*/*z*.

NanoLC-MS/MS analyses were performed using an Easy-nLC 1000 Proxeon chromatographer coupled to LTQ Orbitrap XL (Thermo Fisher Scientific, US-MA) mass spectrometer. Peptide mixtures, dissolved in 2% TFA, were injected onto a capillary chromatographic system consisting of a 2-cm-long trapping column (C18, ID 100 µm, 5 µm, Thermo Fisher Scientific) and a 20-cm-long C18 reverse-phase silica capillary column (ID 75 µm, 5 µm, Nanoseparations). A chromatographic separation based on a gradient lasting overall 125 min was used for peptide fractionation, employing a 250 nL/min flow rate of the following acetonitrile-based eluents: solvent A (0.2% formic acid, 2% acetonitrile LC-MS-grade in water) and solvent B (0.2% formic acid, 95% acetonitrile LC-MS-grade). MS analysis was performed in data-dependent acquisition (DDA) method with MS scans range from 300 to 1800 *m*/*z* followed by isolation and CID fragmentation of the five most intense MS ions (+2,+3,+4 charged) with a dynamic exclusion window of 40 s.

The acquired LC-MS/MS data were further processed with the software Xcalibur v4.2.47. The extracted ion chromatogram (XIC) was obtained for each ion, and each respective area was normalized by using a specific histone non-modified peptide as internal standard. In particular, for H4-deriving peptides, each extracted ion area was divided for the area of peptides 46–55, while for peptides genereting from H3, the reference species was the area of 57–63 peptide ion. TSA/DMSO ratios were obtained by dividing the normalized areas for each species in the respective condition.

### 5.3. Mouse Embryonic Stem Cells (ES14)

Mouse embryonic stem cells, ES14, were grown on 01% gelatin in DMEM high glucose (Gibco, Thermo Fischer Scientific) supplemented with 15% fetal bovine serum (Gibco), 0.1 mM 2(β)-mercaptoethanol (Sigma Aldrich), 1 mM NEAA (Gibco), 2 mM L-glutamine (Gibco), and 1000 units/mL leukemia inhibitory factor (Lif) and grown at 37 °C with 5% CO_2_ in a humidified incubator. Then the ES14 cells were treated with 0.1% DMSO and 100 nM TSA for 24 h. Live phase-contrast images were acquired using a Nikon Eclipse microscope.

### 5.4. RNA Extraction and qPCR Analysis

Total RNA isolation and qPCR analysis have been performed as previously described [42,43]. The following primers were used for *Oct4* amplification (forward) 5′-CCGTGTGAGGTGGAGTCTGGAGAC-3′ and (reverse) 5′- CGCCGGTTACAGAACCATACTCG-3′; *Pdx1* amplification (forward) 5′- GCTCACCTCCACCACCACCTTCC-3′ and (reverse) 5′- GGGTCCTCTTGTTTTCCTCGGG-3′; Gapdh amplification (forward) 5′-AATGGTGAAGGTCGGTGTG-3′ and (reverse) 5′- GAAGATGGTGATGGGCTTCC -3′. Each sample was run in triplicate and normalized to the expression of the housekeeping (Gapdh) gene. Statistical significance between groups was assessed by Student’s t-test. Data are expressed as means ± standard deviation (SD). All experiments were repeated at least three times. A *p*-value < 0.05 was considered to be statistically significant.

### 5.5. Histone Extraction

Core histones were extracted from ES14 cells by performing a fractionated lysis. ES14 cells were firstly resuspended in a hypotonic lysis buffer (10 mM Hepes, 10 mM KCl, 0.1 mM EDTA 0.5 mM PMSF, 0.5% Nonidet-P40 (NP-40), proteases cocktail inhibitors) in a 1:40 *v*/*v* ratio and were left for 15 min on ice, 10 min on a rotator at 4 °C. Finally, the samples were centrifuged for 5 min at 2000 rpm, cytosolic extracts were discarded, and nuclei pellets were recovered and washed with Dulbecco’s phosphate buffer saline (DPBS) and centrifuged for 20 min at 3750 rpm at 4°C. Supernatants were removed, and nuclei were resuspended in DPBS and 0.8 M HCl solution, put on a wheel at 4 °C for 5 h, and then centrifuged for 10 min at 4 °C and 13,200 rpm. The histone’s core was extracted in 0.4 M HCl and dialyzed by 3500 Molecular Weight Cut-Off (MWCO) membranes against 0.1 M acetic acid. Finally, histones core samples were dried in a vacuum SpeedVac system and then resuspended in water.

### 5.6. Densitometric Analysis

Known amounts (1, 2, 4, and 8 µg) of chicken core histones were loaded on a 15% acrylamide/bis-acrylamide SDS-PAGE gel (Supplementary Figure S2). Each histone of the core was separated, and each band was quantified by densitometry analysis by using Quantity One 1-D Analysis Software v4.6.8 (Biorad, Hercules, CA, USA). Each band intensity was associated with the relative amount of core histone, allowing a calibration curve construction for each core subunit. The histone’s core extracted from ES14 was fractionated by SDS-PAGE (Supplementary Figure S3), and each Coomassie-stained band was quantified by densitometry analysis as described for the standard preparation. By interpolating the OD (optical density) of each band with the respective calibration curve, the relative amount of each extracted histone species was determined, and, as a consequence, the trypsin amount to be used for in situ digestion was calculated, with respect to the E:S ratio defined on standard proteins.

## Figures and Tables

**Table 1:** Results of LC-MS/MS analysis of H4 1–17 peptide: post-translational modifications (PTMs), *m*/*z* signal, calculated and theoretical molecular weight (MW), retention time (RT), and peptide fold change (FC) are reported for each species.

	PTMs	*m*/*z*	Calculated MW (Da)	Theoretical MW (Da)	RT (min)	FCs (TSA/DMSO)
TSA	Ac N-ter	806.98	1611.94	1611.93	26.33	0.05
DMSO	Ac N-ter	806.98	1611.94	1611.93	25.48	
TSA	Ac N-ter K16ac	827.98	1653.94	1653.94	28.59	0.34
DMSO	Ac N-ter K16ac	827.98	1653.94	1653.94	27.66	
TSA	Ac N-ter K8ac	827.98	1653.94	1653.94	29.10	0.45
DMSO	Ac N-ter K8ac	827.98	1653.94	1653.94	28.20	
TSA	Ac N-ter K12ac	827.98	1653.94	1653.94	28.28	0.73
DMSO	Ac N-ter K12ac	827.98	1653.94	1653.94	28.45	
TSA	Ac N-ter K5acK16ac	848.98	1695.95	1695.95	31.21	3.28
DMSO	Ac N-ter K5acK16ac	848.98	1695.95	1695.95	30.33	
TSA	Ac N-ter K8acK16ac	848.98	1695.95	1695.95	31.72	2.12
DMSO	Ac N-ter K8acK16ac	848.98	1695.95	1695.95	30.84	
TSA	Ac N-ter K5acK8acK16ac	869.99	1737.96	1737.96	34.94	24.28
DMSO	Ac N-ter K5acK8acK16ac	869.99	1737.96	1737.96	34.12	
TSA	Ac N-ter K5acK12acK16ac	869.99	1737.96	1737.96	35.67	20.56
DMSO	Ac N-ter K5acK12acK16ac	869.99	1737.96	1737.96	34.81	
TSA	Ac N-ter K8acK12acK16ac	869.99	1737.96	1737.96	37.05	54.51
DMSO	Ac N-ter K8acK12acK16ac	869.99	1737.96	1737.96	36.25	
TSA	Ac N-ter K5acK8acK12acK16ac	890.99	1779.97	1779.97	39.02	66.31
DMSO	Ac N-ter K5acK8acK12acK16ac	890.99	1779.97	1779.97	38.26	

**Table 2:** Results of LC-MS/MS analysis of H3 9–17 peptide: post-translational modifications (PTMs), *m*/*z* signal, calculated and theoretical molecular weight (MW), retention time (RT), and peptide fold change (FC) are reported for each species.

	PTMs	*m*/*z*	Calculated MW (Da)	Theoretical MW (Da)	RT (min)	FCs (TSA/DMSO)
TSA	/	451.2642	900.51	900.51	18.75	0.18
DMSO	/	451.2649	900.51	900.51	20.01	
TSA	K9me	458.2720	914.53	914.53	19.28	0.14
DMSO	K9me	458.2714	914.53	914.53	20.50	
TSA	K9me2	465.2796	928.55	928.55	19.57	0.15
DMSO	K9me2	465.2796	928.55	928.55	20.84	
TSA	K9me3	472.2879	942.56	942.56	19.46	0.16
DMSO	K9me3	472.2867	942.56	942.56	20.74	
TSA	K9ac	472.2689	942.52	942.52	21.49	1.90
DMSO	K9ac	472.2705	942.52	942.52	23.47	
TSA	K14ac	472.2692	942.52	942.52	22.07	3.16
DMSO	K14ac	472.2687	942.52	942.52	24.06	
TSA	K9acK14ac	493.2743	984.54	984.54	25.44	28.16
DMSO	K9acK14ac	493.2735	984.54	984.54	27.75	
TSA	K9meK14ac	479.2776	956.54	956.54	22.59	2.68
DMSO	K9meK14ac	479.2765	956.54	956.54	24.56	
TSA	K9me2K14ac	486.2887	970.56	970.56	22.88	4.72
DMSO	K9me2K14ac	486.2847	970.56	970.56	24.88	

## Data Availability

Raw data are available on request that should be send to: montimar@unina.it.

## Supplementary Materials

The following are available online at https://www.mdpi.com/1422-0067/22/4/2063/s1, Figure S1: Coomassie Blue-stained 15% SDS-PAGE of 4 µg of standard chicken core histone (wells 1–9). Each histone band is reported. M: molecular weight size markers. Figure S2: Coomassie Blue-stained 15% SDS-PAGE of 1 µg (wells 1 and 5), 2 µg (wells 2 and 6), 4 µg (wells 3 and 7), 8 µg (wells 4 and 8) of standard chicken core histone. Each histone band is reported. M: molecular weight size markers. Figure S3: Coomassie Blue-stained 15% SDS-PAGE fractionation of acid extraction of ES14 core histones grown in TSA and DMSO conditions. M: molecular weight size markers. Table S1A MALDI-MS and LC-MS/MS data of peptides tryptic mixture from H4 chicken histone, Table S1B: MALDI-MS and LC-MS/MS data of peptides tryptic mixture from H3 chicken histone, Table S2A: Total peptide table of LC-MS/MS analysis of H4 histone, Table S2B: Total peptide table of LC-MS/MS analysis of H3 histone.
