# Supplementary material for: Lysines Acetylome and Methylome Profiling of H3 and H4 Histones in Trichostatin A—Treated Stem Cells
Source: Int J Mol Sci. 2021 Feb 19;22(4):2063. doi: 10.3390/ijms22042063 (PMC7921975; doi:10.3390/ijms22042063)
Supplement: Supplementary file 1 [file ijms-22-02063-s001.zip › Supplementary Figures.pdf]

Supplementary Figures

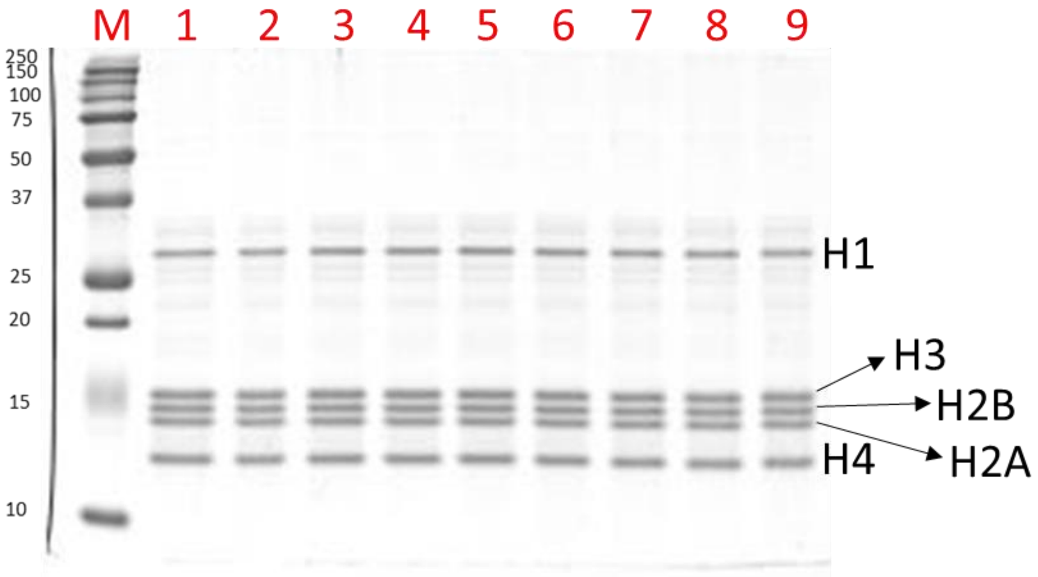

Supplementary Figure S1. Coomassie Blue-stained 15% SDS-PAGE of 4µg of standard chicken core histone (wells 1-9). Each histone band is reported. M: molecular weight size markers.

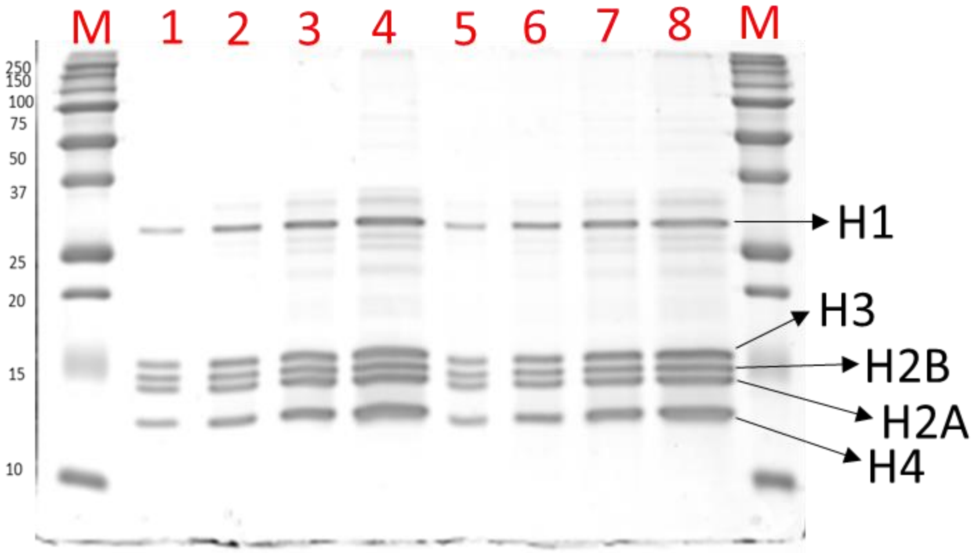

Supplementary Figure S2. Coomassie Blue-stained 15% SDS-PAGE of 1 µg (wells 1 and 5), 2 µg (wells 2 and 6), 4 µg (wells 3 and 7), 8µg (wells 4 and 8) of standard chicken core histone. Each histone band is reported. M: molecular weight size markers

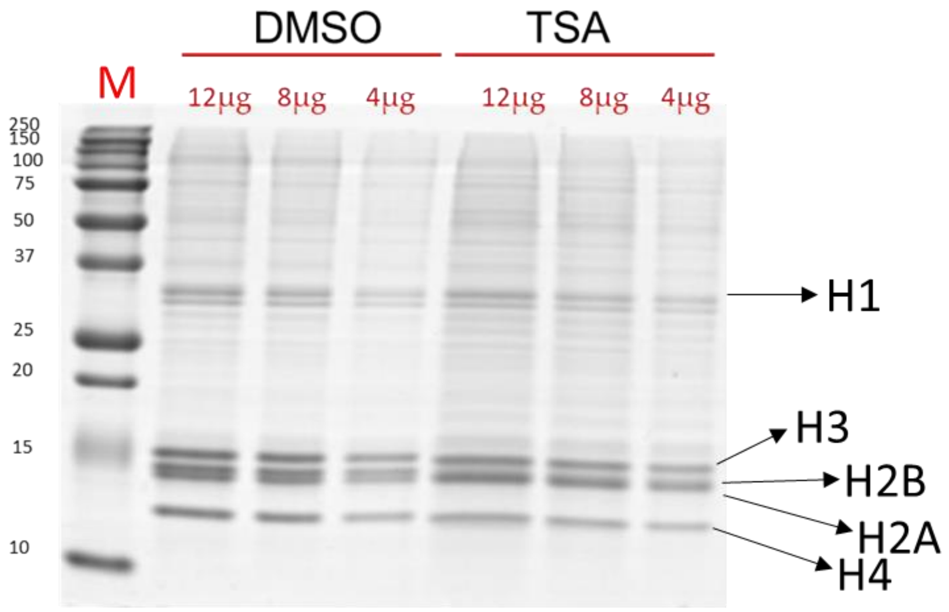

Supplementary Figure S3. Coomassie Blue-stained 15% SDS-PAGE fractionation of acid extraction of ES14 core histones grown in TSA and DMSO conditions. M: molecular weight size markers.
